# Characterizing Chinese undergraduate students’ empathizing-systemizing profiles: a person-centered approach

**DOI:** 10.3389/fpsyg.2024.1395560

**Published:** 2024-07-15

**Authors:** Yishu Qin, Da-Wei Zhang

**Affiliations:** ^1^School of Educational Sciences, Yangzhou University, Yangzhou, China; ^2^Department of Psychology, Jeffrey Cheah School of Medicine and Health Sciences, Monash University Malaysia, Selangor, Malaysia

**Keywords:** empathizing, systemizing, latent profile analysis, cultural adaptation, individual differences

## Abstract

While the empathizing-systemizing (E-S) theory provides a valuable framework for explaining gender differences in STEM majors, previous studies suffer from methodological issues (i.e., the arbitrary cut-off criteria and WEIRD sampling) as well as discrepancies in the behavioral correlates of E-S types. To address the gaps, this study utilized a 3-step latent profile analysis to identify naturally occurring E-S profiles in a Chinese sample and explored the predictors and distal outcomes of the identified profiles. The study recruited 785 (aged 18–25 years, 60% female) Chinese undergraduates. Results revealed five E-S profiles: *Disengaged, Empathizers, Navigating systemizers, Technological systemizers, and Self-declared allrounders*. Controlling for socioeconomic status, being male predicted a higher likelihood of membership into the *Technological systemizers*. Besides, membership to the *Navigating systemizers* and *Technological systemizers* was associated with better intuitive physics performance. However, no significant variation was observed for social sensitivity performance across E-S profiles. Overall, our results partially conformed to previous findings, highlighting the importance of cultural adaptation and methodological considerations when classifying students’ cognitive types.

## 1 Introduction

### 1.1 The E-S theory

To survive in the complex social and physical worlds, we human beings are thought to have evolved two core domains of cognition: empathizing and systemizing (Baron-Cohen, 2009). The domains appear to have distinct biological underpinnings and provide a framework to elucidate individual differences in everyday life (Greenberg and Baron-Cohen, 2020). Empathizing is the drive and ability to identify mental states in others and to give appropriate responses (Baron-Cohen and Wheelwright, 2004; Billington et al., 2007). It encompasses both cognitive and affective components. The cognitive component involves recognition of others’ thoughts and feelings; the affective component involves sharing the feelings of others and enables socially appropriate responses (Greenberg et al., 2018). In contrast, systemizing is the drive and ability to analyze and construct systems from various domains (Baron-Cohen et al., 2003; Billington et al., 2007). It can be a technical system (e.g., the workings of machines), a natural system (e.g., the process of coastal erosion), or even a taxonomic system (e.g., a criterion for ordering books). Typically, we use empathizing to maintain good interpersonal relationship in the social world and use systemizing to understand non-agentive movement in the physical world.

Baron-Cohen and his colleagues suggested that people can be classified according to their abilities to empathize and systemize. These abilities can be measured using the self-reported Empathizing Quotient (EQ) (Baron-Cohen and Wheelwright, 2004) and the Systemizing Quotient (Baron-Cohen et al., 2003). Individuals with higher EQ than SQ (E > S) are classified as *empathizers* or ‘type E’, while the ones who got higher SQ than EQ (S > E) are classified as *systemizers or* ‘type S’. Individuals with relatively equal scores on both EQ and SQ (E = S) can be accounted as ‘type B’ (balanced). Empathizers are believed to have a strong ability of perspective taking, greater ease to feel compassion, and are more comfortable with socializing, while systemizers tend to have strong logical thinking, and might not prefer socializing with others but staying on their own analyzing patterns of physical objects instead.

### 1.2 Gender, major subject, socioeconomic status and the E-S types

As a theory of individual differences, The E-S theory has been applied to explain the gender gap in STEM (Science, Technology, Engineering, and Mathematics) fields. The E-S theory hypothesized a link between gender, major subject choices and the E-S types. It assumes that women and men have different neurological basis that may drive more women toward empathizing and more men toward systemizing (Baron-Cohen et al., 2005). Previous studies consistently reported that women on average were more likely to be classified as empathizers (E > S) and men were more likely to be systemizers (S > E), as measured by questionnaires (Greenberg et al., 2018; Kidron et al., 2018; Rajab et al., 2021). As such, women who have on average a stronger empathizing cognitive tendency can be more inclined to choose ‘people-oriented’ non-STEM subjects (e.g., psychology and literature), while men who have on average a stronger systemizing cognitive tendency are more inclined to choose ‘thing-oriented’ STEM subjects (e.g., physics and engineering) (Manson and Winterbottom, 2012; Svedholm-Häkkinen and Lindeman, 2016; Kidron et al., 2018).

Beyond gender and major choice, socioeconomic status (SES) can be another predictor of the E-S types. Numerous studies have reported lower cognitive performance in relation to unfavorable environment (Liu and Li, 2023). A lower SES can affect one’s brain development and result in smaller volumes of gray matter, which further influence their general cognitive performance (Bignardi et al., 2024). Though the direct link between SES and the E-S types is underexamined, evidence showed an association between the SES, gender and spatial skill, which can be seen as a core systemizing skill. Males from middle- and high-SES backgrounds were found outperformed their female counterparts on spatial tasks, whereas males and females from a low-SES group did not differ in their performance level on spatial tasks (Levine et al., 2005). Regarding the link between SES and empathy, previous studies revealed interesting correlations between SES and different types of empathy. Varnum et al. (2015) found that individuals from higher SES background tended to self-report higher trait empathy, yet their neural empathic responses to faces expressing pain were actually weaker than their counterparts from lower SES background, suggesting that those higher in status may not realize they were actually lower in empathy. Many other empirical studies also replicated the advantage of empathetic responses for individuals from lower SES background in different cultural contexts (Manstead, 2018; Liu et al., 2023). Hence, it would be intriguing to also investigate the link between SES and the E-S types.

### 1.3 Limitations of the conventional E-S taxonomy

Though the E-S theory has been tested and supported in many existing studies, it still suffers from several issues including a) methodological issues of profiling; b) sampling issues; and c) issues with its behavioral outcomes. Firstly, the current E-S types are classified based on D score, which is the difference (D) between the standardized SQ score and the standardized EQ score. Such scoring represents the relative strength of E to S and implies a trade-off between EQ and SQ (Svedholm-Häkkinen and Lindeman, 2016). Yet many studies have shown that the two dimensions tend to be independent in the normal population and high EQ and SQ may not be exclusive of each other (Nettle, 2007; Escovar et al., 2016). In addition, the cut-off criterion for different types is arbitrary and changeable. Some researchers use percentile rank and some use standard deviation from means as cut-off point. For example, Kidron et al. (2018) defined those who scored from lowest to 35*^th^* percentile are Type E while Svedholm-Häkkinen et al. (2018) defined those who scored 1 SD above mean are Type E. Further, current classification masks the variations within EQ and SQ. Both empathizing and systemizing are multifaceted constructs and individuals can also differ on the subfactors of each construct. The EQ is comprised of three subfactors including the *cognitive empathy*, *emotional empathy* and *social skills* (Muncer and Ling, 2006) and the SQ is comprised of four subfactors including the *technicity*, *navigation*, *Do-It-Yourself (DIY)*, and *structure analysis* (see 2.2.2 for detailed explanation) (Ling et al., 2009). It is very likely that one who is classified as a “systemizer” (S > E) according to the conventional E-S taxonomy can actually possess intact cognitive and emotional empathy but cannot display it because she or he is simply unfamiliar with the social rules and does not know how to respond with appropriate manners. Therefore, a new E-S taxonomy uncovering the nuanced variations within the EQ and SQ is needed to further our understanding about individual differences in E-S cognitive tendencies.

Secondly, most research on the E-S theory are based on samples from Western, Educated, Industrialized, Rich and Democratic (WEIRD) societies. The conclusions derived from these WEIRD samples may become maladaptive or “weird” once moved to other cultures (Thalmayer et al., 2021). Cross-cultural studies reported inconsistent results about EQ scores among individuals between eastern Asian countries (e.g., China, Korea, Japan) and their Western counterparts. Reviewing the current literature on the EQ revealed that the average EQ scores of both males and females in Asian countries (for both student and community samples) are roughly one standard deviation lower compared to Western countries, and also the gender differences in these Asian countries are only small in effect size (and not always significant for the total EQ scale) (Groen et al., 2015). However, Eichbaum et al. (2023) pointed out that the idea of unidimensional empathy in intercultural settings may mask the nature of the multifacet construct of empathy as well as the complex context of different cultures. Given that in Western countries it is much more desired to openly express one’s emotion than in Asian countries, empathy may therefore be expressed to a lesser extent in social situations, and gender differences in the inner emotional life may therefore be underestimated or less well recognized when completing the EQ. Other than that, eastern Asian countries that are deeply rooted in Confusionism also emphasizes rules and order, which may further influence the systemizing thought patterns of all citizens in these communities (Ma and Tsui, 2015). Hence, we cannot ignore the unique culture idiosyncrasies reflected in cognitive styles and behaviors among people from different cultures and an in-depth examination of the E-S theory among Eastern culture is necessary.

Thirdly, cognitive E-S propensity and behavioral E-S performance do not always converge. Though most studies on E-S types used self-report measures to gauge the degree of balance between the E and S drives (Naor-Ziv et al., 2021), studies utilized performance tasks often reveal varied results. For example, Billington et al. (2007) applied both questionnaires and performance tests to assess gender differences in empathizing and systemizing. They found that self-reported E-S variations only partially reflected in the test performances. Specifically, significant gender gaps were found in both self-reported EQ and SQ, but only significant female advantage was found in empathizing performance, and no gender difference was detected in systemizing performance. Similarly, Brosnan et al. (2014) found that female advantage in empathy was only significant when measured using self-reported questionnaires but not performance task. However, study by Riekki et al. (2018) showed that systemizing cognitive type was positively associated with better performance in the intuitive physics task, suggesting a correspondence between S propensity and S performance. The existing inconsistent evidence indicates that one’s E-S tendencies are not necessarily consistent with E-S performances. Therefore, it would be intriguing to also explore if one’s E-S cognitive propensity would reflect in his or her E-S behavioral performance when examining their E-S cognitive pattern.

### 1.4 Current study

The present study transcended the traditional E-S brain type classification and used a person-centered approach to reveal the empathizing and systemizing cognitive pattern among a sample of Chinese university students. We used a latent profile analysis to identify individuals with similar E-S cognitive patterns based on the subcomponents of EQ and SQ. To our knowledge, it was the first study that created profiles based on subcomponents of EQ and SQ. While it was difficult to predict the exact latent membership, we expected that some of the emergent profiles might match the cognitive types such as empathizers or systemizers documented in previous studies. We believe the new E-S profiling among Chinese population can further our knowledge of the nuanced individual variations of E-S tendencies as well as examine the E-S theory in a different cultural context. As a follow-up to the first goal, we sought to characterize the demographic predictors and the behavioral outcomes of the latent profiles. In line with previous studies, we hypothesized that the gender, major subject and SES will predict the membership into different E-S profiles. Finally, we assess whether and how the E-S cognitive profiles will be related to individuals’ actual E-S behavioral performances. Based on previous studies, we expect that the E-S cognitive propensities are not always congruent with the E-S performances.

## 2 Materials and methods

### 2.1 Participants and procedure

The sample consisted of 785 undergraduate students from one public university in Jiangsu province, eastern China (female *N* = 477, aged 18 – 25). Participants were recruited from different departments, and the rural-urban ratio of the sample was balanced (50.9% registered with rural hukou). *Hukou* is a household registration system in China. Rural hukou can be considered a proxy for relatively low socioeconomic status because there exists a clear economic gap between rural and urban regions, and rural residents have restricted access to city schools or medical facilities in more productive urban regions in China (OECD, 2017). A detailed breakdown of student characteristics by academic field can be found in Table 1.

**TABLE 1:** Student characteristics by academic field.

Academic field	No. of participants	Female (%)	Rural hukou (%)
Humanities	108	63.9	37.9
Social sciences	171	86.5	48.5
Life sciences	141	68.1	48.9
Natural sciences	180	29.4	60.0
Engineering	178	62.4	53.4

The study was reviewed and approved by the faculty ethics committee. After obtaining consent from the gatekeepers of different departments, we spread a QR code linking to the survey website to students during their evening self-study class (i.e., a popular class in Chinese schools and universities for students in the same major to do homework together from 19:00 to 21:00 in assigned classroom). All measures were completed online via www.wenjuan.com, a reliable Chinese online survey platform similar to Qualtrics. Questionnaires and tests assessing empathizing and systemizing were group administered via smartphones or tablets. To ensure data quality, 17 participants who failed to correctly answer the manipulation check question and 259 participants whose page timing were too long (more than 45 min) or too short (less than 18 min) were excluded from the final sample (Buchanan and Scofield, 2018). Students were told that their participation was completely voluntary and that their answers would be kept confidential.

### 2.2 Measures

#### 2.2.1 Empathizing Quotient—Short

Empathizing drive was assessed using the 15-item self-reported Empathizing Quotient – Short (EQ-S) scale (Muncer and Ling, 2006). The EQ-S is comprised of three subfactors: cognitive empathy, emotional empathy, and social skills. Cognitive empathy items focus on understanding the intentions and emotions of others (5 items; e.g., “I am good at predicting how someone will feel”). Emotional empathy items focus on feeling or sharing the affective states of others (5 items; e.g., “Seeing people cry really upset me”). Social skills items focus on the ability to communicate and interact with others (5 items; e.g., “I find it hard to know what to do in a social situation”). Each item scored zero for (strongly or slightly) disagreeing, one for slightly agreeing and two for strongly agreeing with the item (some items are reversely scored). Potential scores ranged from 0 to 30. Reliability of the scale was estimated using McDonald’s Omega (ω), an advantageous estimator of reliability compared to Cronbach’s α because it does not assume essential tau-equivalence (Hayes and Coutts, 2020). The internal consistency for EQ-S was ω = 0.88.

#### 2.2.2 Systemizing Quotient—Short

Systemizing drive was assessed using the 18-item self-reported Systemizing Quotient – Short (SQ-S) scale (Ling et al., 2009) It is comprised of four subfactors: technicity, navigation, Do-It-Yourself (DIY), and structure analysis. Technicity items focus on interest in understanding technical details of mechanical devices (6 items; e.g., “If I were buying a car I would want to obtain specific information about its engine capacity”). Navigation items focus on the assessment of ability to accurately ascertain desired positions or routes (3 items; e.g.,“I find it difficult to learn my way around a new city”). DIY items focus on the interests to make and repair things oneself (3 items; e.g., “If there is a problem with the electrical wiring in my home I would be able to fix it myself “). Structure analysis items focus on interest or understanding of the structure of objects (6 items; e.g., “When I look at a building I am curious about the precise way it was constructed”). SQ-S was scored the same way as the EQ-S, with potential scores ranging from 0 to 36. The internal consistency for SQ-S was ω = 0.94.

#### 2.2.3 Social Sensitivity test

Empathizing performance was measured using a social sensitivity test called the social stories task (Lawson et al., 2004), which evaluate people’s understanding of social outcomes in complex, contextualized social scenarios. It provides a useful index of how individuals apply their 1) understanding of intentional mental states, 2) appreciation of emotional impact of words, and 3) knowledge of social norms to identify socially disrespectful behavior (Meinhardt-Injac et al., 2020). Therefore, it serves as an appropriate instrument to assess empathizing behavior in current study. The social stories task contains 10 short vignettes depicting different social contexts involving utterances among characters. Each vignette falls into three sections, which comprised of 4–6 utterances. Participants were required to identify the faux pas utterance made by one character that could upset another character from the stories. Among 30 sections of the ten stories, ten sections contain a blatant target faux pas, ten contains a subtle target faux pas, and ten contains no target faux pas (see Supplementary Appendix for a sample vignette). Given that very few non-target utterances were mistaken for faux pas, the erroneous identification of the non-targets was not included in this study. The sum of the number of correctly identified faux pas was counted as the final score (ranged 0 - 20), reflecting one’s actual ability to empathize with the others.

#### 2.2.4 Intuitive Physics task

**FIGURE 1:**
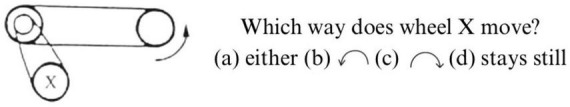
An example question from the intuitive physics test (the correct answer is b).

Systemizing performance was measured using the Intuitive Physics task (Baron-Cohen et al., 2001). The test consists of 20 choice questions, each has a cue picture and multiple outcome options. The cue picture depicts a mobile system with different kinds of objects (e.g., gear, pulley, or pendulum) and an arrow marking the direction of movement of one object (see Figure 1 for an example question). Participants were asked to choose the most likely outcome of a certain object from the moving system as to where the object in the picture will end up. The number of correct answers was summed as the final score (ranged 0 - 20), reflecting one’s actual ability to systemize the mechanical processing of physical objects.

#### 2.2.5 Socioeconomic status

Socioeconomic status was measured using the Family Affluence Scale (FAS; Hartley et al., 2016). The FAS is usually considered an objective measure of SES (Currie et al., 2008) as it gauges the material family wealth as an indicator of the absolute level of socioeconomic position. It is comprised of 4 items: ownership of family car (0,1,2), own bedroom (no = 0, yes = 1), family holidays during the past 12 months (0,1,2,3 or more), and family computer (0,1,2,3 or more). The FAS works especially well with young people still in full-time education and without occupational status given that they do not always know or want to report their parents’ income or education level (Currie et al., 2008). In this case, FAS works as a less intrusive, more comprehensible approach to identify SES. It has been used among Chinese students and showed good reliability and validity (Liu et al., 2012).

All measures applied in the present study were developed originally in English. The Chinese version of the empathizing scale and social stories test were translated and verified in simplified Chinese by scholars from mainland China, therefore was utilized directly in current research. But the Chinese version of the systemizing scale was translated into traditional Chinese by Cheng and Hung of the National Yan-Ming University from Taiwan, China. Given that the characters and wordings of the traditional Chinese used in Taiwan are slightly different from the simplified Chinese used in mainland China, we adapted the systemizing scale into simplified Chinese with the assistance of a PhD student majoring in linguistics. Terms such as “Yin Ti” (硬體), meaning hardware, was adapted to “Yin Jian” (硬件) to make the expressions more idiomatic for participants from mainland China.

The Intuitive physics test was translated into Chinese following the forward-backward translation method (Cha et al., 2007). Two Chinese-English bilingual graduate students first translated the test into Chinese, then another student who had no knowledge of the original version back-translated the test into English again. The researcher compared the back-translated version with the original one to ensure the content and meaning were not distorted or lost in translation. Finally, the Chinese and English versions of all measures were reviewed by six bilingual Chinese students to obtain the final version.

## 3 Analytic strategies

Data analysis proceeded in three steps. We first conduct a preliminary exploratory structural equation modeling to verify the measurement factor structure of the EQ (3 subfactors) and SQ (4 subfactors) (ESEM; Asparouhov and Muthén, 2009). The standardized scores of the verified EQ and SQ subfactors were then used as the indicator for the latent profile analysis (LPA) to identify the E-S cognitive profiles in our sample. Once the optimal profile solution was determined, we incorporated predictors to the model via a multinomial logistic regression analysis using the R3STEP method and examined the statistical significance of outcomes differences across profiles using the BCH method. All latent variable analyses were conducted in Mplus 8.6 (Muthén and Muthén, 1998).

### 3.1 Missing data

Missing values for the items were minimal (ranging from 0.1 – 1.1). Little’s MCAR test revealed that the missing data was completely missing at random (χ^2^(107) = 108.52, *p* = 0.44). Missing values in SPSS 26.0 were imputed using the expectation-maximization algorithm. Missing values in Mplus were handled by the Full Information Maximum Likelihood (FIML), using all available data to maximize the information.

### 3.2 Preliminary measurement models

The factor structure of the EQ and SQ scales was verified in ESEM models. A confirmatory approach to ESEM was adopted so that items were specified to load on their respective factors and cross-loadings were targeted to be as close to zero as possible using target rotation. The ESEM approach was selected because it allows less restrictive measurement models when small cross-loadings were expected among subfactors (Asparouhov and Muthén, 2009). This is the case of the present study where theoretical and empirical association can be expected among some subfactors (e.g., cognitive empathy and emotional empathy; Groen et al., 2015). Model fit was assessed using the comparative fit index (CFI), the root mean square error of approximation (RMSEA), and the standardized root mean-square residual (SRMR). Good model fit is indicated by a CFI value close to 0.9 or above, a RMSEA value close to 0.06 or below, and SRMR close to 0.08 or below (Hu and Bentler, 1999).

### 3.3 Latent profile analyses

Models with 2 to 6 profiles were computed to identify subgroups of individuals who showed similar empathizing-systemizing patterns. Standardized scores from the confirmed subfactors verified in the ESEM model were used as indicators for the latent profile membership. The optimal number of profiles was guided by several statistical criteria (Nylund et al., 2007). The model fit was assessed by the Akaike information criterion (AIC), the Bayesian information criteria value (BIC), the Sample-size adjusted BIC (SaBIC), with lower values indicating a better model fit. The Lo-Menddell-Rubin test (LMRT) and bootstrapped likelihood ratio test (BLRT) were conducted for each solution to compare the k-1 versus k class model, with a non-significant LMRT and BLRT test support a model with one less profile. The entropy value was used to assess classification accuracy, with higher values representing greater precision in classification. It worth noting that statistical simulation studies have shown that some statistical indices (i.e., BIC, ABIC, BLRT) were far more effective than others, and should be prioritized in selecting the optimal number of profiles (Marsh et al., 2009). Besides, the information criteria were plotted in an elbow graph to visualize the cut-off point where the curve begins plateauing. More importantly, the final decision regarding the optimal number of profiles should always be based on the theoretically interpretation ability of the solutions, and not only on the information criteria.

### 3.4 Predictors and outcomes of latent profile membership

The predictors and outcomes of latent profiles were assessed using the auxiliary modeling in Mplus (Asparouhov and Muthén, 2009). To investigate the effect of gender, major subject and SES on profile memberships, the AUXILIARY = X (R3STEP) command was performed to conduct multinomial logistic regressions. Specifically, the latent profile variable, as the dependent variable, was regressed on auxiliary variables, as predictors, simultaneously in the R3STEP model. To examine the distal outcomes of the E-S profiles, the AUXILIARY = Y (BCH) command was performed. The BCH method is conceptually equivalent to a Chi-Square analysis, which examined the significance of differences in social sensitivity and intuitive physics across profiles. Specifically, the latent profile variable was performed as independent variable and the auxiliary variables was performed as dependent variables in the BCH model. Both the R3STEP and BCH methods can avoid shifts in latent profiles and is suitable for both continuous and categorical covariates.

## 4 Results

### 4.1 Empathizing-systemizing profiles identification

The ESEM models with target rotation showed good fit to data (CFI = 0.915, RMSEA = 0.037, SRMR = 0.024), supporting the underlying factor structure of the EQ and SQ constructs. Standardized scores from the confirmed subfactors verified in the ESEM model were then used as indicators for the latent profile identification. Fit indices for the 2- to 6-profile solutions can be found in Table 2. BLRT tests are significant for all solutions, thereby provided limited information to determine the optimal number of profiles. The AIC, BIC and SABIC values were graphically presented as an elbow plot (see the Supplementary Appendix). The 5-profile solution appeared to be the elbow point of the information criteria curve, suggesting that adding another profile no longer give much better modeling of the data. However, given the LMR value of the 5-profile solution was only marginally significant (*p* = 0.08), which indicates that this model might not be superior to solutions with lesser profiles, we then carefully examined it in conjunction with the 3-, 4-, and 6-profile solutions. This examination revealed that adding a fourth and fifth profile to the solution resulted in a theoretically interpretable and qualitatively distinct result, while adding a sixth profile simply split an existing profile into smaller ones with similar pattern. The entropy value kept on increasing as the number of profiles increased from three to five, showing a higher level of classification accuracy with additional profiles. Moreover, the non-significant LMR value (*p* = 0.21) of the six-profile solution also supported a model with one less profile. Thus, based on fit indices and interpretability of the profiles, the five-profile solution was selected.

**TABLE 2:** Model fit indices for the latent profile classification with 2–6 classes.

Profile	AIC	BIC	SABIC	pLMR	pBLRT	Entropy	Group size
2	14929.90	15032.38	14962.52	0.00	0.00	0.76	573/206
3	14820.71	14960.45	14865.18	0.01	0.00	0.71	475/145/159
4	14750.08	14927.08	14806.41	0.22	0.00	0.74	444/156/147/32
**5**	**14689.46**	**14903.72**	**14757.65**	**0.08**	**0.00**	**0.75**	**431/128/119/65/36**
6	14665.37	14916.91	14745.43	0.21	0.00	0.68	298/178/132/97/41/33

Values in bold type is the selected model. AIC, Akaike information criterion; BIC, Bayesian information criteria; SaBIC, Sample-size adjusted bayesian information criteria; LMR, Lo-Menddell-Rubin test; BLRT, Bootstrapped likelihood ratio test.

**FIGURE 2:**
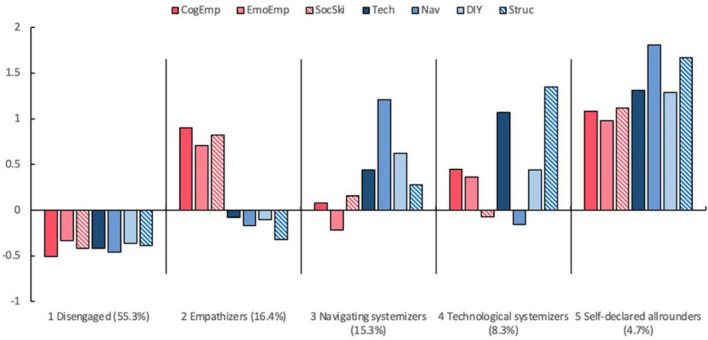
Characteristics of the empathizing-systemizing profiles. CogEmp, cognitive empathy; EmoEmp, emotional empathy; SocSki, social skills; Tech, technology; Nav, Navigation; DIY, Do-It-Yourself; Struc, Structure analysis.

The five empathizing-systemizing profiles were labeled as (1) Disengaged, (2) Empathizers, (3) Navigating systemizers (4) Technological systemizers, and (5) Self-declared allrounders. Scores of each indicator for different profiles were standardized and these profiles are illustrated in Figure 2. Profile 1 was the largest group in this study (55.3%). Individuals in this group showed balanced low empathizing and low systemizing tendencies, with all mean scores below average. Due to their lack of interest in both empathizing and systemizing activities, we labeled this the *Disengaged* profile (Profile 1). Individuals in Profile 2 showed a propensity to empathize. This group scored consistently high on all EQ subscales but low on SQ subscales, conforming to the definition of empathizers (E > S) based on the extreme male brain theory. As such, we referred to them as *Empathizers* (Profile 2), who accounted for 16.4% of the participants. In contrast, individuals in Profile 3 showed a propensity to systemize. This group’s EQ subscale scores were consistently lower than their SQ subscale scores, conforming to the definition of systemizers (S > E) based on the E-S theory. Amongst all SQ subscales, they scored extremely high on Navigation (1.21), showing a strong sense of direction. Also, despite of their relatively low EQ as compared to their own SQ, their cognitive empathy (0.08) and social skills (0.16) were acceptable, whereas their emotional empathy (−0.22) were below average. In other words, this group of people were systemizers who could understand, but were not affected by, others’ feelings. Thereby, instead of using the simple label *systemizers*, we referred to them as *Navigating systemizers* (Profile 3), who accounted for 15.3% of the participants. Individuals in Profile 4 could be distinguished from all other participants by their extreme enthusiasm for electronic gadgets. These people scored especially high on technology (1.07) and structure analysis (1.35), showing a strong tendency to not only use the high-tech products, but also understand how it works. Nevertheless, this group of individuals had little interest in social activities. Despite of their medium level of cognitive empathy (0.45) and emotional empathy (0.36), they remained strange to social rules (−0.07). Also, they displayed poor sense of direction (−0.16), indicating certain unfamiliarity to outdoor activities. To capture the nuanced characteristics of this profile, they were labeled as the *Technological systemizers* (Profile 4) and comprised only 8.3% of participants. Finally, there was a small subset of people who scored extremely high on all EQ and SQ subscales (4.7%). However, their actual performance on social sensitivity and intuitive physics tasks were the worst among all profiles (see section “4.3 Outcomes of profile membership”), which did not conform to their all-round image. In this case, the last group was named as the *Self-declared allrounders*.

### 4.2 Predictors of profile membership

Next, we examined whether gender and major subject were significant predictors of the profile membership while controlling for the effect of SES as covariate (see Table 3). Gender was dummy coded and women served as the reference category. A significant positive coefficient plus an odds ratio (OR) > 1 suggested that, compared to women, men had an increased likelihood of belonging to the target profile (vs. the comparison profile). In line with the E-S theory, men were more likely to be categorized in the *Technological systemizers* profile. Results showed that men were more commonly found in the *Technological systemizers* profile (Profile 4) relative to the *Disengaged*, *Navigating systemizers*, and *Self-declared allrounders* profiles (Profile 1, 3, 5), ORs = 0.26 − 0.46. However, there was no significant effect of major subject in predicting profile memberships. Though not the focus of the present study, we observed significant effect of SES in predicting profile membership. Those from the high-income families had an increased likelihood of being classified *as Technological systemizers* or *Disengaged people* (vs. *Empathizers* or *Navigating systemizers*).

**TABLE 3:** Multinomial logistic regression for the effects of predictors on profile membership.

Predictor	Profile 1 vs. 2		Profile 1 vs. 3		Profile 1 vs. 5		Profile 4 vs. 1		Profile 4 vs. 2	
	Coef.	OR	Coef.	OR	Coef.	OR	Coef.	OR	Coef.	OR
Gender	0.14	1.15	0.16	0.85	0.56	0.57	0.79*	1.46	0.65	0.52
Major	−0.12	0.89	−0.06	0.94	−0.17	0.84	−0.17	0.85	−0.29	0.75
SES	0.54***	1.72	0.46**	1.58	0.80	2.22	0.19	1.21	0.73**	2.07
**Predictor**	**Profile 4 vs. 3**		**Profile 4 vs. 5**		**Profile 3 vs. 2**		**Profile 2 vs. 5**		**Profile 3 vs. 5**	
	**Coef.**	**OR**	**Coef.**	**OR**	**Coef.**	**OR**	**Coef.**	**OR**	**Coef.**	**OR**
Gender	0.95*	1.38	1.35*	1.26	0.31	1.36	−0.71	0.49	−0.40	0.67
Major	−0.23	0.80	−0.34	0.71	−0.06	0.94	−0.05	0.95	−0.11	0.90
SES	0.65*	1.91	0.99	2.68	0.08	1.09	0.26	1.29	0.34	1.40

The logits and odds ratio reflect the effects of predictors on the likelihood of membership into the first listed profile relative to the second listed profile. Profile 1 = Disengaged; Profile 2 = Empathizers; Profile 3 = Navigating systemizers; Profile 4 = Technological systemizers; Profile 5 = self-declared allrounders; SES, Socioeconomic Status; Coef., coefficient; OR, odds ratio. *p < 0.05, **p < 0.01, ***p < 0.001.

### 4.3 Outcomes of profile membership

**FIGURE 3:**
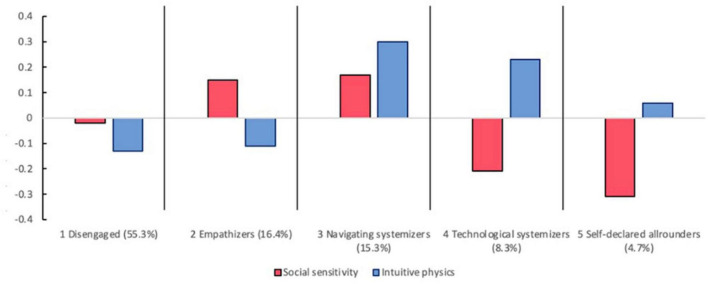
Social sensitivity and intuitive physics task performances across profiles.

The final aim was to investigate differences in empathizing and systemizing performances across the five profiles while controlling for gender, major subject, and socioeconomic status. Profile-specific means for social sensitivity (the indicator for empathizing performance) and intuitive physics (the indicator for systemizing performance) are shown in Figure 3. Regarding the social sensitivity performance, an omnibus test for an overall difference across the profiles was χ^2^ (4) = 6.83, *p* = 0.15, suggesting that profile membership had no significant relation with individuals’ ability to recognize faux pas in social situations.

In contrast, the latent profiles showed significant impact on intuitive physics performance, χ^2^ (4) = 15.15, *p* = 0.004. Pairwise comparisons indicated that *navigating systemizers* obtained the highest scores (0.30) in intuitive physics task and were significantly better than the disengaged and the empathizer group. Next, the *technological systemizers* who showed strong interest in scrutinizing object functional rules had the second-best intuitive physics performance (0.23) and did not differ from *the navigating systemizers* (0.30). As expected, the *disengaged* people (−0.13) and *empathizers* (−0.11) scored lowest in intuitive physics. The *self-declared allrounders* stayed in-between, obtaining a score very close to zero (0.06). These findings suggested that the systemizing performance echoes the self-reported systemizing propensity in this study.

Overall, consistent to their indifference to both empathizing and systemizing activities, the *disengaged people* showed mediocre to poor performances on social sensitivity and intuitive physics. Similarly, despite of their strong confidence in themselves, the *self-declared allrounders* underperformed in social sensitivity and showed mediocre performance in intuitive physics. Consistent with their profile images, *empathizers* outperformed in social sensitivity but underperformed in intuitive physics, whereas the *technological systemizers* displayed reversed pattern. Interestingly, the *navigating systemizers* displayed the best performance not only in intuitive physics but also social sensitivity, suggesting a propensity toward systemizing activities based on pure interest rather than their actual abilities.

## 5 Discussion

The nuanced findings from the present study of Chinese undergraduates illustrated the importance for researchers and educators to consider the multi-dimensional nature of E-S constructs and culture diversity as well as to distinguish between cognitive propensity and behavioral performance when classifying students into certain cognitive types. Using the latent profile analysis, five groups of individuals sharing similar patterns of E-S subfactors were identified and compared to other groups, both in terms of how the E-S subfactors combined to form the groups, and how those combinations are differentially related to E-S performances. The newly classified E-S profiles partially conformed to the E-S types reported in previous studies. Variations within EQ and SQ were captured, portraying a more fine-grained image for each group. Further, gender and SES, but not major subject, were found to be the significant predictors of certain profile memberships. Last but not least, systemizing propensity reflected in systemizing performance, but empathizing propensity did not always reflect in empathizing performance. Significant variations were observed only for intuitive physics task but not social sensitivity test across E-S profiles.

### 5.1 Identified E-S cognitive profiles and behavioral outcomes

Specifically, current study identified five emergent subgroups of individuals with unique E-S patterns (disengaged people, empathizers, navigating systemizers, technological systemizers, self-declared allrounders) and documented each profile’s prevalence. These profiles partially conformed to the existing E-S types in prior studies, suggesting that the profiles identified here are likely to be robust. Profile 1 – the disengaged group (*N* = 431) – represented the largest proportion (55.3%) of the total sample. They showed no interest in either empathizing or systemizing activities. Consistent with their perceived below average E-S propensities, the disengaged group also exhibited weak intuitive physics performance and average social sensitivity. Similar pattern were also found for the Low-Low (i.e., low EQ and low SQ) balanced brain type group reported in a study of three thousand Finnish college students by Svedholm-Häkkinen and Lindeman (2016). The Low-Low Finnish group also scored low in social intelligence and physics test. The consistent findings between the Chinese and Finnish population indicate that the disengaged group were very likely to be robust across culture.

In contrast, Profile 5 – the self-declared allrounders (*N* = 36) reported strong interests in both empathizing and systemizing activities, displaying similar E-S cognitive pattern to the “High-High” (i.e., high EQ and high SQ) balanced brain type group from Finland (Svedholm-Häkkinen and Lindeman, 2016). This group only represented 4.7% of the total sample. However, opposite to their double high E-S tendencies, these people scored the lowest on social sensitivity test and the third on intuitive physics task among all five profiles. Such discrepancy between self-reported tendency and behavioral performance was not found in previous research with Western population. The High-High Finnish group displayed equivalently well-adjusted and satisfied social intelligence and physics grades as their perceived E-S tendencies (Svedholm-Häkkinen and Lindeman, 2016). The discrepancy between strong E-S propensities and mediocre E-S performances among the self-declared allrounders from present study potentially stems from the Chinese culture of Mianzi (i.e., Face). The Chinese concept of “face” is a measure of one’s status, prestige, and social position (Filieri et al., 2017). As the Chinese writer Lin Yutang put: “The psychological face …is not a face that can be washed or shaved, but a face that can be ‘granted’ and ‘lost’ and ‘fought for”’(p.199, Lin, 1935). Given that the Chinese society values social status and interpersonal relationship to a high degree, some people would aggrandize themselves in all aspect to “fight for their face.” The small group of allrounders in current study were likely to overestimate their E-S to maintain a positive image among others. However, we should not rule out the chance that these self-declared allrounders were simply passionate about all kinds of E-S activities regardless of their actual capacities. Further studies were required to investigate whether or not this profile rated their E-S propensities extremely high on purpose.

Profile 2 – the empathizers showed strong tendency to empathize with others but weak tendency to systemize. This group of people correspond to the characteristics of the Type E (E > S) reported in previous studies (Wakabayashi et al., 2007; Kidron et al., 2018) and represented 16.4% of the total sample. Moreover, their high E - low S propensity also reflected in their performance, displaying strong social sensitivity but weak intuitive physics. Similar correspondence between E-S propensity and performance were also found for the British (Lawson et al., 2004) and Finnish empathizers (Svedholm-Häkkinen and Lindeman, 2016), indicating a cross-cultural consistency in the cognitive and behavioral E-S patterns of the empathizers.

Finally, current study identified two types of systemizers. Both Profile 3 - the navigating systemizers (15.3%) - and Profile 4 - the technological systemizers (8.3%) - demonstrated stronger tendency to systemize than to empathize. However, the low E - high S propensity only reflected in performance for the technological systemizers. The navigating systemizers outperformed all other profiles in both social sensitivity and intuitive physics tasks. A closer look at the EQ and SQ subfactors revealed that the navigating systemizers were extremely confident in their sense of direction, which could indicate good mental rotation, that is the ability to simulate 3D-rotation of objects in one’s mind (Riekki et al., 2018). Given that the intuitive physics task requires also mental simulation or in other words imagining in one’s mind how things move, the outstanding performance in intuitive physics of navigating systemizers were very likely attributed to their mental rotation capacity (Mitko and Fischer, 2020). Surprisingly, despite of their self-reported average level of cognitive empathy and even below-average level of emotional empathy, the navigating systemizers demonstrated satisfactory ability to identify faux pas that may upset others in conversations in the social sensitivity test. One possibility is that they had underestimated their empathy. Though they showed good absolute level of social sensitivity, they could still have relatively low interest in social interactions as compared to their interest in physical systems. Another possibility is that the navigating systemizers had applied their systemizing ability to “systemize empathy,” therefore bypassed the actual empathizing approach to achieve high scores in social sensitivity test. Put differently, instead of using the empathizing skills such as perspective-taking or emotion-sharing, the navigating systemizers might utilized their systemizing skills to grasp social rules to analyze different social situations (Golan and Baron-Cohen, 2006; Valla et al., 2010). On the contrary, the low E - high S propensity in the technological systemizers corresponded to their performance, showing strong intuitive physics but weak social sensitivity. This group of individuals portrayed an image that conformed to the “nerd” or “geek” stereotype (Tintori and Palomba, 2017). They could be distinguished from all other profiles by their passion for technology but indifference to social activities. Although they reported adequate level of perceived empathy, their actual performance on social sensitivity test was below average. On the contrary, their familiarity about latest technology and extreme enthusiasm for understanding the structures of mechanic products helped them to make causal inferences about moving physical objects to tackle questions from the intuitive physics test.

To sum up, evidence from current study revealed nuanced E-S profiles. Specifically, we identified two types of systemizers: one with better navigation skill and one with better technicity and structure analysis skills. Furthermore, our results also suggest that individuals’ cognitive E-S propensities do not always reflect in their E-S performances among Chinese undergraduates The disengaged group, empathizers group and technological systemizers group showed E-S performance that consistent with their E-S propensities, but the self-declared allrounders and navigating systemizers’ empathizing performances were not consistent with their self-reported empathizing propensities. Future research is required to explore why such discrepancy happened for different groups of people.

### 5.2 Gender and other predictors of the E-S profiles

As theoretically hypothesized in the E-S theory, the present study was consistent with previous research in which men showed stronger propensity as well as performance to systemize than women (Kidron et al., 2018). Compared with women, men were more likely to be in the technological systemizers group than in the disengaged, self-declared allrounders and navigating systemizers groups. However, we did not find significant link between E-S profiles and subject choices, which differs from previous findings with students from western, educated, industrialized, rich, and democratic (WEIRD) countries. Prior studies with WEIRD students consistently reported that systemizers tend to choose STEM subjects such as Physics and Engineering, while empathizers tend to choose non-STEM subjects such as English Literature and Sociology (Corrêa Varella et al., 2016; Groen et al., 2018). Such phenomenon was not found for Chinese students. Unlike their WEIRD peers who enjoy well-developed social welfare systems and could prefer their major subjects according to interests or strengths, many Chinese students may choose their major subjects for utilitarian reasons. Studies of attitude toward science showed that students from developing countries believed that science can “make their lives healthier, easier and more comfortable” and were more aspired to study STEM subjects (Sjøberg and Schreiner, 2012). It is very likely that students from low SES families may select STEM subjects regardless of their cognitive tendencies to earn a good life in the future. Current study found that participants majored in STEM indeed reported lower family affluence level than those majored in humanities and social sciences. Future studies could explore the association between major subject and E-S profiles in the rich and poor students separately. Furthermore, we found that SES could significantly predict the membership of technological systemizers group. This could be attributed to the fact that children from high SES families have more chance to access latest technology.

Although this study creates a new taxonomy of E-S profiles and contributes knowledge of E-S performances in each profile as well as their associations with gender, major subject and socio-economic status, several limitations could be addressed in future research. First, current study utilized only college students from one Chinese university, the generalizability of the profiles as well as the relationship between the profiles and predictors/outcomes warrant additional investigation. Future studies could extend the study with larger and more diverse samples. Our findings indicate that self-reported empathizing and systemizing are culture-sensitive and people from different cultural backgrounds who reported the same E-S propensities could display different E-S performances. It is important to utilize cultural theories to investigate how culture affect E-S tendencies and behaviors. For example, the individualism-collectivism theory proposes that Westerners prioritize individual goals and see themselves as autonomous agents whilst Asians prioritize group goals and see themselves as being fundamentally interconnected and defined by their relationship with others (Kitayama, 2002; Steele and Lynch, 2013). It is possible that the individualism versus collectivism cultural differences influence people’s self-perceptions about their empathizing and systemizing. Future studies could therefore focus on the mechanism of how culture shapes empathizing and systemizing by including measures of individualism and collectivism cultural values. In addition, from a developmental perspective, there may be age-related changes in how people construct their empathizing and systemizing. For instance, Greenberg et al. (2018) reported that age was positively correlated with both EQ and SQ. Nevertheless, Beadle and de la Vega (2019) found that older adults had lower cognitive empathy but higher emotional empathy than younger adults. As a result, a longitudinal study that identifies E-S profiles across multiple time points could reveal intriguing changes in people’s E-S patterns. Lastly, it is worth noting that we did not include the distractor items in the EQ and SQ to prevent social desirable responding, which may add to the discrepancy between the self-reported and actual test performance. Also we used the brief SQ which may have poorer validity than the SQ-Revised which is the official version. Besides, there are other behavioral tests for empathizing and systemizing which tap into different facets of these two constructs. For example, Reading the Mind in the Eyes Test is another widely used performance task for empathy (Greenberg et al., 2023). It can gauge people’s ability to identify emotions from only the eye region of faces. Future studies could include a series of performance tasks to uncover more nuanced relationship between E-S propensities and performances.

## 6 Conclusion

Previous research that has studied empathizing and systemizing cognitive styles classified people into different brain types based on the discrepancy between EQ and SQ, rendering variations within empathizing and systemizing invisible. Moreover, only a few studies have deployed a variable-oriented approach to examine the correlations between E-S propensities and performances. To unpack the variability within empathizing and systemizing, the present study used the person-centered approach of a 3-step latent profile analysis to search for new E-S taxonomy among Chinese college students. Five E-S profiles were identified and labeled as (1) Disengaged, (2) Empathizers, (3) Navigating systemizers, (4) Technological systemizers, and (5) Self-declared allrounders, partially conforming the traditional E-S brain types. Further, unlike previous findings among WEIRD sample, only gender and SES, but not subject major, were correlated with E-S profiles. Finally, we found that the E-S propensities were not always reflected in E-S performances. Only systemizing performance, but not empathizing performance, was significantly different between the E-S cognitive profiles. Overall, this study builds upon and extends the predominantly Western research on E-S cognitive styles in a Chinese context, highlighting the importance to consider cultural effect as well as the distinction between cognitive propensities and behavioral outcomes when classifying students into different types.

## Data availability statement

The raw data supporting the conclusions of this article will be made available by the authors, without undue reservation.

## Ethics statement

The studies involving humans were approved by the Ethics Committee of the School of Educational Sciences, Yangzhou University. The studies were conducted in accordance with the local legislation and institutional requirements. Written informed consent for participation was not required from the participants or the participants’ legal guardians/next of kin because the research involves no more than minimal risk to the subjects.

## Author contributions

YQ: Conceptualization, Data curation, Formal analysis, Funding acquisition, Investigation, Methodology, Software, Writing – original draft, Writing – review and editing. D-WZ: Data curation, Validation, Writing – review and editing.
