# Supplementary material for: Characterizing Chinese undergraduate students’ empathizing-systemizing profiles: a person-centered approach
Source: Front Psychol. 2024 Jul 15;15:1395560. doi: 10.3389/fpsyg.2024.1395560 (PMC11284084; doi:10.3389/fpsyg.2024.1395560)
Supplement: Supplementary file 1 [file Data_Sheet_1.docx]

**Supplementary Materials**

**Appendix 1.** A sample vignette from the social stories task (as the social sensitivity test)


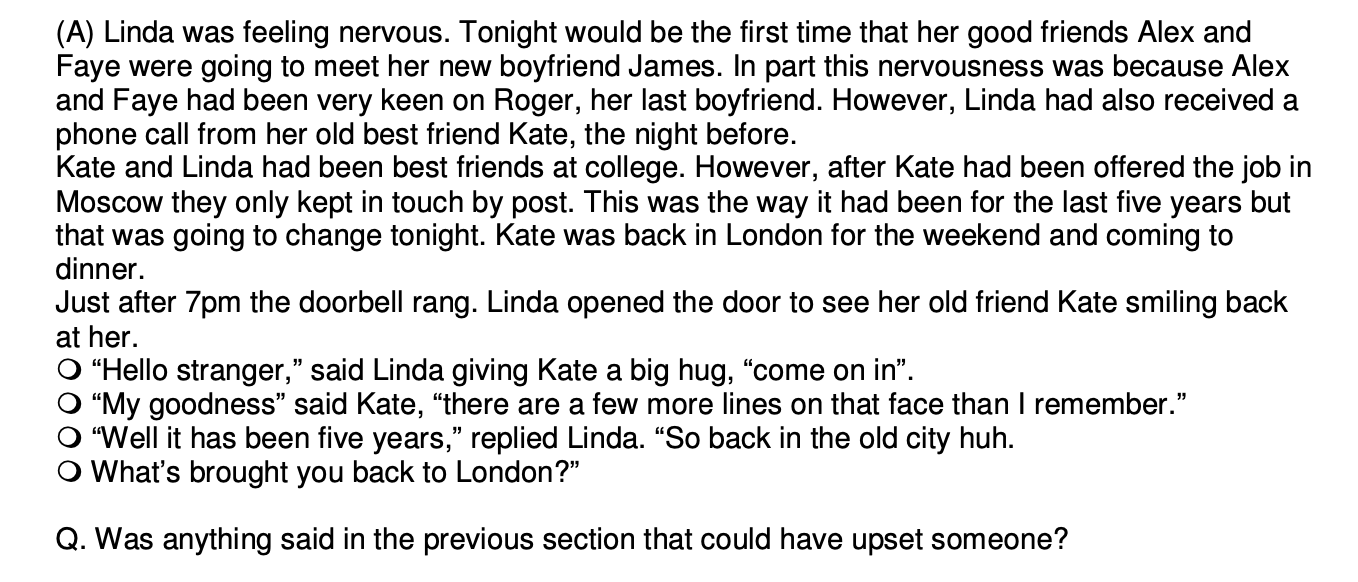


The correct answer is conversation Line 2 “My goodness” said Kate, “there are a few more lines on that face than I remember”. It is a blatant faux pas which may upset the protagonist Linda.

**Appendix 2.** Elbow plots for latent profile analyses

**
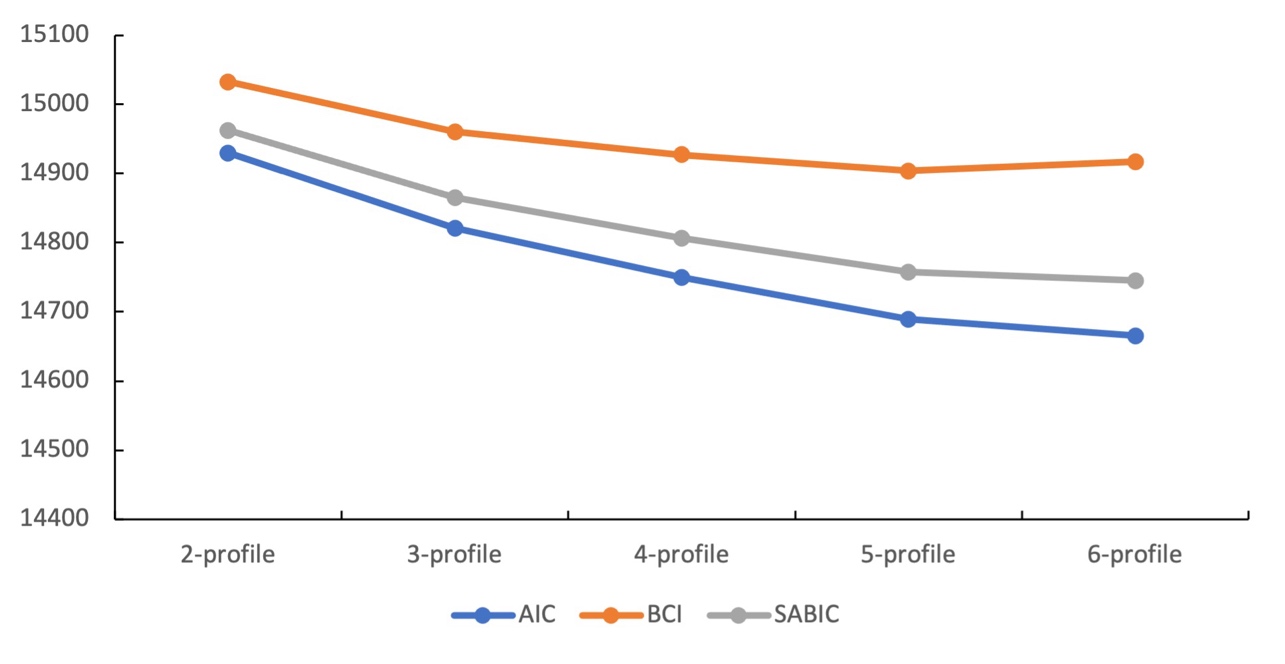
**
